# Refining early detection of Marburg Virus Disease (MVD) in Rwanda: Leveraging predictive symptom clusters to enhance case definitions

**DOI:** 10.1016/j.ijid.2025.107902

**Published:** 2025-07

**Authors:** Olivier Nsekuye, Frederick Ntabana, Hugues Valois Mucunguzi, Ziad El-Khatib, Eric Remera, Lyndah Makayotto, Menelas Nkeshimana, David Turatsinze, Frederic Ntirenganya, Semakula Muhammed, Athanase Rukundo, Brian Chirombo, Richard Muvunyi, Claude Mambo Muvunyi, Pacifique Nizeyimana, Yvan Butera, Sabin Nsanzimana, Edson Rwagasore

**Affiliations:** 1 Rwanda Biomedical Centre, Kigali, Rwanda; 2 Department of Global Public Health, Karolinska Institutet, Stockholm, Sweden; 3 World Health Organization, Kigali, Rwanda; 4 Rwanda Ministry of Health, Kigali, Rwanda; 5 The University Teaching Hospital of Kigali (CHUK), Kigali, Rwanda; 6 Rwanda Development Board (RDB), Kigali, Rwanda; 7 Adventist University of Central Africa (AUCA), Kigali, Rwanda

**Keywords:** Marburg Virus Disease, Symptom patterns, Early detection, Machine learning, Outbreak response

## Abstract

**Background:**

Marburg Virus Disease (MVD) poses a significant global health risk due to its high case fatality rates (24%-88%) and the diagnostic challenges posed by its nonspecific early symptoms, which overlap with other febrile illnesses like malaria. This study analyzed symptom patterns from the 2024 MVD outbreak in Rwanda to refine case definitions and enhance early detection.

**Methods:**

A retrospective analysis was conducted of 6613 suspected MVD cases (66 positive, 6547 negative) reported between September 27 and December 20, 2024. Symptom prevalence and predictive value were assessed using multiple logistic regression models with L1 and L2 regularization to identify the most predictive symptoms. Models were validated using 5-fold cross-validation, with performance assessed through ROC analysis and standard accuracy metrics.

**Results:**

Fever (78.8%), fatigue (63.6%), and headache (57.6%) were identified as the most common early symptoms, while hemorrhagic signs were rare (3.0%). The model achieved high accuracy (99.04%) and an area under the receiver operating characteristic curve of 0.824, identifying fever, fatigue, nausea/vomiting, joint pain, and sore throat as key predictors.

**Conclusion:**

Early symptom clusters, especially constitutional and gastrointestinal signs outperformed hemorrhagic symptoms for MVD detection. Findings challenge current case definitions, emphasizing the need for revised public health messaging and healthcare worker training. Integrating symptom-based models into surveillance could enhance detection, especially in resource-limited settings.

## Highlights

• Early MVD symptoms are mostly constitutional; hemorrhagic signs are rare (3%).
• Machine learning model predicts MVD with 99.04% accuracy from symptom patterns.
• Fever plus fatigue and nausea/vomiting form key early diagnostic cluster.
• Focus on hemorrhagic symptoms in case definitions may delay detection.

## Introduction

Marburg Virus Disease (MVD), a highly fatal illness caused by infection with the Marburg virus or Ravn virus, is a current public health challenge, considering its mortality complex clinical presentation, and lack of approved medical countermeasures (MCMs)—MCMs such as vaccines and therapeutics. MVD was first identified in 1967 during simultaneous outbreaks in Germany and Yugoslavia; cases have since been reported primarily in sub-Saharan Africa, with case fatality rates ranging from 24% to 88%, depending on outbreak context and healthcare resources. Since then, significant outbreaks have been documented across Africa: Angola (2004-2005, 374 cases, 88% fatality), Democratic Republic of Congo (1998-2000, 154 cases, 83% fatality), Uganda (multiple outbreaks in 2007, 2012, 2014, and 2017 with fatality rates ranging from 27% to 100%), Ghana (2022, 4 cases, 75% fatality), Equatorial Guinea (2023, 16 confirmed cases plus 23 probable cases, with nearly 100% fatality among confirmed cases), and Tanzania (2023, 9 cases, 67% fatality). Additionally, isolated cases have been reported in travelers visiting caves inhabited by fruit bats in Uganda (cases in the Netherlands and USA, 2008), as well as sporadic cases in Kenya and South Africa. These outbreaks demonstrate the virus's geographic spread, potential for international travel-associated cases, and varying case fatality rates across different healthcare contexts [1,2].

Early diagnosis is critical for outbreak control but is challenged by nonspecific initial symptoms, including fever, headache, and fatigue, which overlap with other endemic febrile illnesses. As the disease progresses, symptoms such as unexplained bleeding, jaundice, and multi-organ failure may emerge; however, these distinctive symptoms are not universally present, complicating timely case identification. This clinical variability underscores the urgent need for refined, evidence-based diagnostic frameworks to guide frontline healthcare workers during early outbreaks [3,4].

Although it is highly recommended that during viral hemorrhagic fever (VHF) outbreaks, including MVD, a more conservative approach should be taken to prevent additional spread in the community. Current diagnostic approaches integrate epidemiological exposure history with broad clinical criteria to improve sensitivity. However, this approach often sacrifices specificity, leading to over-diagnosis, unnecessary isolation, and resource strain. In regions where malaria and typhoid fever are endemic, this diagnostic ambiguity can overwhelm healthcare systems and delay containment efforts [5].

The recent Marburg outbreak in Rwanda declared on September 27, 2024, resulted in 66 confirmed cases with 15 deaths (22.7% case fatality rate). This outbreak highlighted the variability in clinical presentations. While some patients may develop hemorrhagic manifestations, consistent with the classification of the disease as a VHF, many patients in this outbreak presented with minimal or no bleeding signs. This spectrum of clinical presentations underscores the need for case definitions that capture the full range of MVD manifestations [6]. The Marburg virus is primarily transmitted through direct contact with the bodily fluids of infected individuals, including blood, vomit, urine, and feces. The virus can also spread through contact with contaminated materials and surfaces. The Egyptian fruit bat, *Rousettus aegyptiacus* of the *Pteropodidae* family, is now well-established as the natural reservoir host of the Marburg virus. Several outbreaks have been linked to human exposure in mines or caves harboring these bat colonies [7].

Human-to-human transmission occurs primarily through direct contact with infected bodily fluids during three high-risk activities: providing care for infected individuals at home, healthcare settings where infection control precautions are inadequate, and traditional funeral practices involving direct contact with deceased victims. Healthcare workers are at particular risk when appropriate personal protective equipment and infection control measures are not strictly enforced. Sexual transmission has also been documented, with viral persistence in seminal fluid for up to 7 weeks after clinical recovery, necessitating abstinence or protected sexual activity during this period. There is no evidence of airborne transmission of the Marburg virus in natural settings, although laboratory-acquired infections have raised concerns about potential aerosol transmission in certain contexts [5,8].

Diagnosis of MVD presents significant challenges, particularly in early disease stages. Laboratory confirmation is achieved through several methods: reverse transcription polymerase chain reaction (RT-PCR) for detecting viral RNA, antigen-capture enzyme-linked immunosorbent assay (ELISA) for viral proteins, IgM and IgG antibody ELISAs, serum neutralization testing, electron microscopy, and virus isolation by cell culture. RT-PCR is most useful in early infection, while antibody testing becomes valuable later. Currently, treatment is primarily supportive, focusing on symptom management, maintaining fluid and electrolyte balance, and addressing complications such as hemorrhage, shock, and secondary infections. Intravenous fluid replacement, pain management, antiemetics, and treatment of coinfections may significantly improve survival rates. While several experimental therapeutics (including monoclonal antibodies, antivirals, and immunomodulators) and vaccine candidates have shown promise in animal models and early clinical trials, none are yet approved for routine clinical use. This emphasizes the importance of early detection, isolation, and supportive care as the cornerstone of outbreak control [2,5,8].

Diagnosis of MVD faces several significant challenges that impact outbreak response and patient management. First, early clinical presentation often mimics more common endemic diseases such as malaria, typhoid fever, shigellosis, meningitis, and other VHF, making initial clinical diagnosis difficult. Second, laboratory infrastructure is limited in many endemic regions, with few facilities meeting the biosafety level 4 requirements necessary for virus isolation. Third, serological tests may show cross-reactivity with other filoviruses, potentially complicating definitive diagnosis. Fourth, viral load varies significantly depending on the disease stage, with detection becoming challenging in very early or late phases of infection. Fifth, point-of-care diagnostic options remain limited in resource-constrained settings, delaying confirmation and appropriate isolation measures. Finally, the sporadic and often rural nature of outbreaks means diagnostic capacity is rarely prepositioned where cases first emerge. These limitations emphasize the need for robust clinical case definitions, enhanced surveillance systems, and rapid deployment of field diagnostic capabilities during suspected outbreaks [1,2].

Emerging data analysis techniques, particularly machine learning (ML), provide novel opportunities to enhance diagnostic frameworks. ML algorithms can analyze complex outbreak data to identify and rank predictive patterns of disease presentation, providing actionable insights for early case identification [9]. This study used ML methods to analyze Rwanda's outbreak data, identifying the most predictive and observable symptoms of MVD. By bridging theoretical diagnostic frameworks with field application, our findings aim to strengthen outbreak preparedness, improve early diagnosis, and support containment efforts during future outbreaks.

## Methods

### Study design and setting

**Figure 1:**
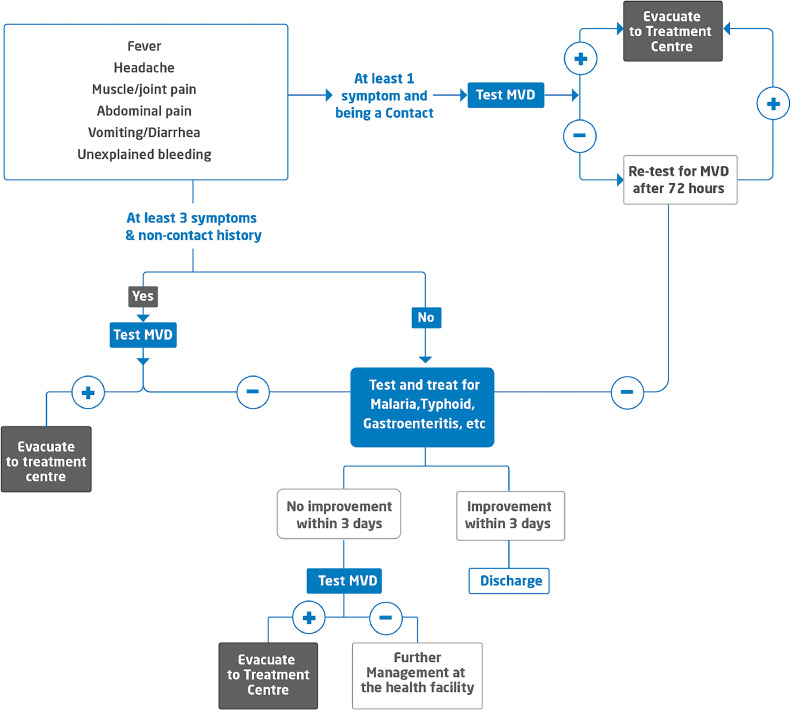
Marburg testing algorithm in Rwanda.

This retrospective study analyzed clinical data from the 2024 MVD outbreak in Rwanda, which occurred between September 27 and December 20, 2024. The outbreak was concentrated in Kigali City, where confirmed cases were managed at designated treatment centers, including Nyamata Treatment Center and Baho Treatment Center. The diagnosis followed Rwanda's standardized testing algorithm, coordinated by the Rwanda Biomedical Center (RBC), which ensured screening and diagnosis using RT-PCR for suspect cases and contacts of confirmed cases who developed symptoms during the 21-day follow-up period, as guided by the case definition (Figure 1).

The suspect case definition (Figure 1) was developed by the Rwanda Ministry of Health in consultation with the World Health Organization (WHO), and US CDC experts, adapting the WHO's standard case definition to the local context while maintaining core surveillance criteria.

### Study population and data collection

The study included all individuals who were tested for MVD during the outbreak period (September 27 to December 20, 2024) and who met the suspect case definition as outlined in Figure 1. Inclusion criteria were: 1) presence of at least one symptom consistent with the case definition, 2) completion of clinical and epidemiological data collection, and 3) availability of RT-PCR test results. Individuals were excluded if they had incomplete symptom data that could not be reliably imputed or if their samples were deemed inadequate for testing. A total of 6613 individuals met the inclusion criteria and were included in the analysis.

The study included individuals tested for MVD who met the suspect case definition (Figure 1), encompassing both RT-PCR-confirmed positive and negative cases. Clinical and epidemiological data were recorded in the MVD electronic system during the outbreak. Collected variables included demographic details (age, gender, occupation, residence), symptom profiles (onset, duration, severity), and laboratory RT-PCR results. Symptom verification involved cross-checking patient-reported data with healthcare workers’ clinical observations during triage and follow-up.

### Data analysis

#### Feature selection

The analysis aimed to identify the most predictive symptoms of MVD. The dependent variable was confirmed MVD diagnosis, coded as a binary outcome (1 = confirmed case, 0 = negative case). Independent variables included symptoms such as fever, unexplained bleeding, nausea/vomiting, diarrhea, jaundice, muscle pain, joint pain, intense fatigue, sore throat, encephalopathy, conjunctivitis, difficulty breathing, cough, abdominal pain, bloody diarrhea, headache, skin rash, anorexia, chest pain, flu-like symptoms, and difficulty swallowing.

While many symptoms aligned with the official suspect case definition, additional symptoms were included based on clinical reports and patient records, capturing atypical or less common manifestations of MVD. This comprehensive approach ensured evaluation of the full spectrum of clinical presentations. Each symptom was encoded as a binary variable (1 = present, 0 = absent).

Feature selection proceeded in two stages. First, exploratory data analysis assessed symptom prevalence, co-occurrence patterns, and correlations using frequency distributions and heat maps to identify common clusters (e.g., fever with fatigue and headache). Second, logistic regression with regularization ranked symptoms by predictive importance. L1 regularization (Lasso) was applied to eliminate less relevant features by shrinking coefficients to zero, while L2 regularization (Ridge) minimized collinearity and stabilized coefficient estimates.

#### Data processing

Data processing involves multiple steps to ensure a clean and robust dataset. Missing symptom data for noncases were imputed using multiple imputations, leveraging symptom distribution patterns among confirmed cases to reduce bias and maintain completeness. Symptom variables were encoded as binary indicators (1 = present, 0 = absent) for uniformity. The dataset was split into training (70%) and test (30%) subsets; the training set was used for model development and validation, while the test set provided an independent assessment of model performance.

#### ML models

Logistic regression with regularization was the primary modeling approach, chosen for its balance of interpretability and predictive power. The model estimated the likelihood of MVD diagnosis based on symptom profiles. L1 regularization (Lasso) performed feature selection by shrinking coefficients of nonpredictive symptoms to zero, while L2 regularization (Ridge) mitigated overfitting and addressed multicollinearity.

Five-fold cross-validation optimized the regularization parameter (*λ*), balancing model complexity and performance. Two key *λ* values were considered: *λ*min, which minimized binomial deviance for maximum accuracy, and *λ*_1se_, which favored a simpler model with marginally lower accuracy but improved robustness, and interpretability.

#### Model performance evaluation

Model performance was assessed using standard diagnostic metrics: 1) Accuracy: proportion of correctly classified cases; 2) sensitivity: ability to identify true positives (confirmed MVD patients); 3) specificity: ability to exclude false positives (non-MVD cases); 4) additionally, the area under the receiver operating characteristic curve (AUC-ROC): the area under the ROC, reflecting the model's discriminative ability to distinguish between MVD-positive and negative cases. A higher AUC indicated superior model performance.

#### Software and tools

All analyses were performed using R and relevant statistical packages. Logistic regression with regularization (L1 and L2) was implemented using the *glmnet* package, providing a robust framework for feature selection and model evaluation. Feature importance was visualized with the *vip* (Variable Importance Plot) package, offering insights into symptom contributions to MVD diagnosis.

Data cleaning and preprocessing were conducted using *dplyr* and *tidyr,* ensuring a standardized and high-quality dataset for analysis. Visualizations, including heat maps and coefficient plots, were generated using *ggplot2*, enhancing the interpretability and clarity of results.

#### Outcome measures

The primary outcome was the identification of the most predictive symptoms for MVD diagnosis, ranked by importance in the logistic regression model. These findings aim to inform the development of evidence-based, symptom-driven diagnostic criteria for practical use in field settings. Secondary outcomes included insights into symptom co-occurrence patterns identified during exploratory analyses. These patterns may contribute to refining broader diagnostic strategies and improving the understanding of MVD's diverse clinical manifestations.

### Ethical considerations

This study was conducted under the approval of the Ministry of Health/Rwanda Biomedical Centre (RBC) as part of an emergency response to the Marburg outbreak in Rwanda. Given the retrospective nature of the analysis using de-identified data collected during a standard outbreak response, individual patient consent was waived. All data handling followed strict confidentiality protocols, with analysis conducted on secured systems with restricted access.

## Results

### Symptoms prevalence and distribution among MVD suspected cases

The clinical profile of 6613 suspected cases, including 6547 negative and 66 positive cases, revealed notable differences in symptom prevalence (Table 1). Among confirmed cases, fever was the most common symptom, observed in 52/66 (78.8%) compared to 3359/6547 (51.3%) negative cases. Intense fatigue or lethargy was reported in 42/66 (63.6%) positive cases versus 1990/6547 (30.4%) negative cases, while headache was reported in 38/66 (57.6%) compared to 2948/6547 (45.0%).

**Table 1:** Symptom prevalence among suspected MVD cases during the Rwanda outbreak, 2024.

	Total (*N* = 6613)	Negative (*N* = 6547)	Positive (*N* = 66)
**Fever**			
No	3202 (48.4%)	3188 (48.7%)	14 (21.2%)
Yes	3411 (51.6%)	3359 (51.3%)	52 (78.8%)
**Intense fatigue/lethargy**			
No	4581 (69.3%)	4557 (69.6%)	24 (36.4%)
Yes	2032 (30.7%)	1990 (30.4%)	42 (63.6%)
**Nausea/vomiting**			
No	5371 (81.2%)	5334 (81.5%)	37 (56.1%)
Yes	1242 (18.8%)	1213 (18.5%)	29 (43.9%)
**Joint pain**			
No	5580 (84.4%)	5536 (84.6%)	44 (66.7%)
Yes	1033 (15.6%)	1011 (15.4%)	22 (33.3%)
**Headache**			
No	3627 (54.8%)	3599 (55.0%)	28 (42.4%)
Yes	2986 (45.2%)	2948 (45.0%)	38 (57.6%)
**Diarrhea**			
No	5711 (86.4%)	5657 (86.4%)	54 (81.8%)
Yes	902 (13.6%)	890 (13.6%)	12 (18.2%)
**Muscles pain**			
No	5556 (84.0%)	5507 (84.1%)	49 (74.2%)
Yes	1057 (16.0%)	1040 (15.9%)	17 (25.8%)
**Chest pain**			
No	6217 (94.0%)	6153 (94.0%)	64 (97.0%)
Yes	396 (6.0%)	394 (6.0%)	2 (3.0%)
**Abdomen/stomach pain**			
No	5338 (80.7%)	5288 (80.8%)	50 (75.8%)
Yes	1275 (19.3%)	1259 (19.2%)	16 (24.2%)
**Cough**			
No	6313 (95.5%)	6251 (95.5%)	62 (93.9%)
Yes	300 (4.5%)	296 (4.5%)	4 (6.1%)
**Anorexia/loss of appetite**			
No	5479 (82.9%)	5430 (82.9%)	49 (74.2%)
Yes	1134 (17.1%)	1117 (17.1%)	17 (25.8%)
**Sore throat**			
No	6471 (97.9%)	6407 (97.9%)	64 (97.0%)
Yes	142 (2.1%)	140 (2.1%)	2 (3.0%)
**Difficult breathing**			
No	6452 (97.6%)	6388 (97.6%)	64 (97.0%)
Yes	161 (2.4%)	159 (2.4%)	2 (3.0%)
**Difficult swallowing**			
No	6468 (97.8%)	6402 (97.8%)	66 (100%)
Yes	145 (2.2%)	145 (2.2%)	0 (0%)
**Bloody diarrhea**			
No	6431 (97.2%)	6367 (97.3%)	64 (97.0%)
Yes	182 (2.8%)	180 (2.7%)	2 (3.0%)
**Jaundice/yellow eyes/gum skin**			
No	6570 (99.3%)	6504 (99.3%)	66 (100%)
Yes	43 (0.7%)	43 (0.7%)	0 (0%)
**Conjunctivitis/red eyes**			
No	6546 (99.0%)	6482 (99.0%)	64 (97.0%)
Yes	67 (1.0%)	65 (1.0%)	2 (3.0%)
**Flu**			
No	6467 (97.8%)	6401 (97.8%)	66 (100%)
Yes	146 (2.2%)	146 (2.2%)	0 (0%)
**Skin rash**			
No	6547 (99.0%)	6482 (99.0%)	65 (98.5%)
Yes	66 (1.0%)	65 (1.0%)	1 (1.5%)
**Unexplained bleeding**			
No	6519 (98.6%)	6455 (98.6%)	64 (97.0%)
Yes	94 (1.4%)	92 (1.4%)	2 (3.0%)
**Encephalopathy**			
No	6613 (100%)	6547 (100%)	66 (100%)

Other key symptoms among positive cases included nausea/vomiting (29/66; 43.9%) and joint pain (22/66; 33.3%). Symptoms such as diarrhea (18.2%), muscle pain (25.8%), and abdominal pain (24.2%) were moderately prevalent. Rare symptoms included chest pain, sore throat, and bloody diarrhea (each 3.0%). Notably, difficulty swallowing, jaundice, and flu-like symptoms were absent among confirmed cases, while unexplained bleeding (3.0%) and skin rash (1.5%) occurred infrequently.

### Symptoms co-occurrence among MVD-positive confirmed cases

**Figure 2:**
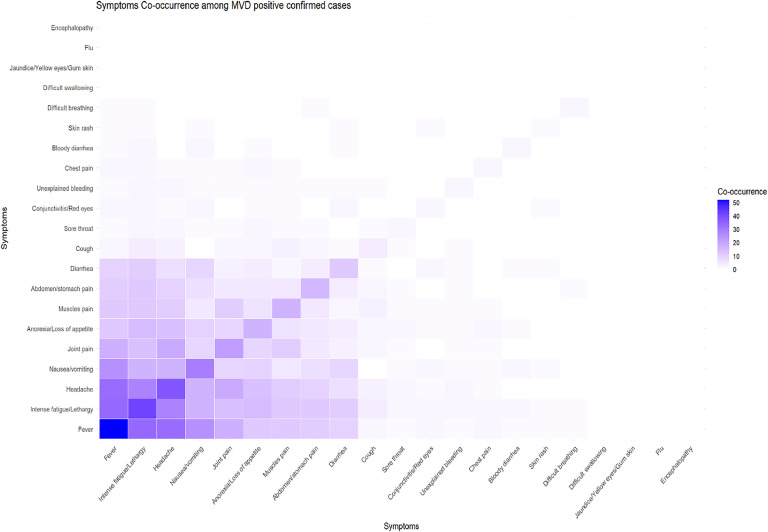
Heatmap of symptoms co-occurrence among MVD-positive confirmed cases.

A heatmap (Figure 2) illustrates symptom co-occurrence patterns among confirmed MVD cases, with darker shades indicating higher frequencies of co-occurrence. Fever was the most prominent symptom, frequently co-occurring with intense fatigue/lethargy, headache, and nausea/vomiting, forming a core cluster of symptoms. Moderate co-occurrence was observed for joint pain, loss of appetite/anorexia, muscle pain, abdominal pain, and diarrhea, supporting their association with MVD. In contrast, symptoms such as encephalopathy, flu-like symptoms, jaundice, and difficulty swallowing showed limited co-occurrence, suggesting they are less central to the clinical profile of MVD.

### Predictive importance of symptoms in diagnosing MVD

**Figure 3:**
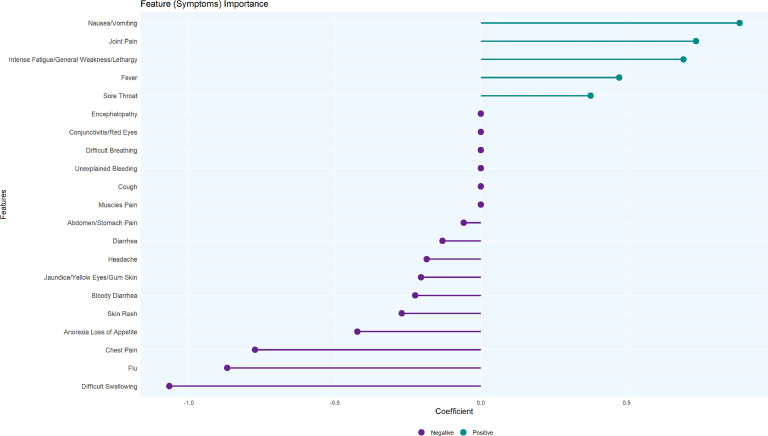
Coefficients of symptoms in logistic regression model.

Figure 3 illustrates the logistic regression coefficients with regularization, illustrating the relative importance of symptoms in predicting MVD diagnosis. Positive coefficients (turquoise bars) indicate symptoms that increase the likelihood of MVD, while negative coefficients (purple bars) represent symptoms less associated with confirmed cases.

Key predictors with strong positive associations included nausea/vomiting, joint pain, intense fatigue, fever, and sore throat, suggesting their importance in MVD diagnosis. Conversely, symptoms such as difficulty swallowing, flu-like symptoms, and chest pain had significant negative coefficients, highlighting their lower relevance in distinguishing MVD cases from noncases.

The logistic regression model's performance was robust, with using cross-validation optimizing the regularization parameter (*λ*) to achieve a achieve a balance between accuracy and interpretability (*λ*min = 0.000880). The model demonstrated excellent discrimination with an AUC-ROC of 0.824, reflecting its strong ability to differentiate between positive and negative cases. The overall accuracy was 99.04%, with a misclassification rate of 0.96%, confirming the model's reliability.

## Discussion

This study provided a comprehensive analysis of the 2024 MVD outbreak in Rwanda, challenging traditional diagnostic assumptions and offering insights to enhance early detection strategies. It underscored the importance of recognizing early symptom clusters to improve outbreak control.

The predominance of fever, intense fatigue, and headache reflects the fundamental pathophysiology of filovirus infections, driven by systemic inflammation and cytokine release during early viral replication. Similar findings were reported during the 2007 Uganda outbreak [5]; however, this study adds statistical rigor through ML approaches. Notably, the significant prevalence of gastrointestinal symptoms such as nausea and abdominal pain may indicate viral tropism for the gastrointestinal epithelium. Prior studies [10,11] have shown viral proteins compromise intestinal barrier function, making these symptoms critical for differentiating MVD from other febrile illnesses.

The low prevalence of hemorrhagic symptoms in this cohort has significant implications for surveillance and case definitions. Hemorrhagic manifestations, linked to late-stage endothelial injury and coagulopathy, were rare and occurred late in disease progression [10,12]. Over-reliance on hemorrhagic features for diagnosis, as seen during the 2005 Angola outbreak [13], risks delayed case identification and subsequent disease spread^.^

Co-occurrence analysis highlighted key symptom clusters—fever, fatigue, headache, nausea, joint pain, muscle pain, and abdominal pain—as reliable early indicators of MVD. This finding aligns with the Tanzanian study [14] that emphasized recognizing symptom patterns rather than isolated symptoms for early detection.

Predictive modeling revealed a distinct symptom profile strongly associated with confirmed MVD cases, including fever, nausea/vomiting, joint pain, intense fatigue, and sore throat. These findings are consistent with the pathophysiological progression of filovirus infections, where systemic inflammatory responses trigger both constitutional and organ-specific symptoms [10]. Similar patterns were observed during the 2014 Democratic Republic of Congo outbreak [15], but our study added robust statistical validation via ML. These insights have significant implications for current screening protocols. The strong predictive value of constitutional and gastrointestinal symptoms underscores the need to revise case definitions to capture early disease presentations. In resource-limited settings, recognizing early symptom clusters can guide triage and isolation, reducing early symptom clusters can guide triage and isolation, reducing nosocomial transmission, and improving outbreak containment [16].

The practical implementation of our findings suggests a resource-optimized approach to case management during outbreaks, guided by the symptom clusters identified through our analysis. Patients presenting with the complete predictive cluster (fever with fatigue/headache plus gastrointestinal symptoms like nausea/vomiting) would warrant immediate high-level isolation and expedited testing based on their elevated probability of MVD. Those with partial presentations (such as fever with either headache or gastrointestinal symptoms alone) would still receive testing but could be managed in intermediate isolation areas with regular reassessment as symptoms evolve. Importantly, our approach does not advocate for excluding any suspected cases from testing but rather for allocating resources proportionally to clinical probability while maintaining comprehensive surveillance. This stratified approach, derived directly from our symptom importance analysis, could improve resource utilization during outbreaks while maintaining sensitivity for case detection. Implementation would require standardized assessment protocols and validation during future outbreaks.

The ML model's ability to identify predictive symptoms highlights its potential utility in resource-limited settings. However, implementation must consider dataset imbalances and real-world complexities, as noted in a 2019 study [17]. Public health messaging and healthcare worker training must also evolve. Current emphasis on hemorrhagic symptoms may delay care-seeking behavior among patients presenting with nonhemorrhagic, early-stage symptoms. Furthermore, healthcare workers in endemic regions, where febrile illnesses are common, require additional training to recognize these early predictive symptoms and ensure immediate laboratory diagnosis to differentiate MVD from other endemic conditions.

A critical limitation of our study is that patients sought medical care at different time points in their disease progression, creating a mixed picture of MVD at various stages rather than at standardized time points. As symptoms evolve throughout the course of illness, our identified patterns may reflect this temporal variability rather than a consistent clinical profile, challenging our ability to establish definitive early-stage diagnostic criteria. Additional limitations include the retrospective design, which may introduce documentation bias, and the single-country focus, which limits generalizability. Future research should prioritize prospective, multi-country studies that capture patients at more uniform disease stages to validate these findings across diverse contexts.

In conclusion, this study identified key early symptom clusters for MVD detection, with constitutional and gastrointestinal signs outperforming hemorrhagic symptoms. The model achieved high accuracy (99.04%) and an AUC-ROC of 0.824, identifying fever, fatigue, nausea/vomiting, joint pain, and sore throat as primary predictors. These findings challenge current case definitions, emphasizing the need for revised public health messaging and healthcare worker training that focuses on early, nonhemorrhagic presentations. Integrating predictive symptom-based models into surveillance systems could significantly enhance early detection capabilities, especially in resource-limited settings where rapid identification is crucial for outbreak containment.

## Declarations of competing interest

The authors declare that they have no known competing financial interests or personal relationships that could have appeared to influence the work reported in this article.
